# Energy-efficient data collection optimization for dual-UAV-assisted relay marine networks

**DOI:** 10.1038/s41598-025-10766-9

**Published:** 2025-07-10

**Authors:** Fukang Deng, Haibo Luo, Furong Xu, Ming Li, Jianshan Zhang

**Affiliations:** 1 School of Computer and Big Data, Minjiang University, Fuzhou, 350108 China; 2 College of Computer and Data Science, Fuzhou University, Fuzhou, 350118 China; 3 Fujian Institute of Education, Fuzhou, 350025 China

**Keywords:** Energy-Efficient, UAV Trajectory, Data Offloading, Resource Allocation, Marine Network, Mathematics and computing, Computational science, Computer science

## Abstract

With the rapid development of the marine economy, the conflict between the limited capacity of existing network infrastructure and the growing demand for marine data collection has become increasingly prominent. Due to their high mobility and ease of deployment, unmanned aerial vehicles (UAVs) are playing an increasingly important role in marine data collection and transmission. This paper investigates the marine data collection problem with dual-UAV-assisted relay. Specifically, it aims to minimize the total system energy consumption while satisfying constraints such as quality of service and service coverage, through joint optimization of access relationship, UAV trajectory, data offloading, and communication resource allocation. To address this non-convex mixed-integer nonlinear programming problem, a joint optimization algorithm is proposed. The algorithm first employs a greedy algorithm-based approach to solve the access relationship optimization sub-problem, then applies a successive convex approximation (SCA)-based approach to solve the UAV trajectory and communication resource allocation sub-problems, and finally iteratively solves these sub-problems to obtain a near-optimal solution to the original problem. Simulation results demonstrate that, compared with several benchmark algorithms, the proposed joint optimization algorithm significantly reduces the total system energy consumption while ensuring the successful completion of all data collection.

## Introduction

The marine environment, which spans more than 70% of the Earth’s surface^1^, serves as a critical reservoir of diverse resources for humanity and plays a pivotal role in global climate regulation. The global expansion of the marine industry has resulted in a substantial increase in the number of ships utilized for marine transportation, tourism, fisheries, and other economic activities. This development has coincided with a significant escalation in the demand for effective monitoring and sustainable management of the marine environment^2,3^. Therefore, the collection and utilization of marine data have become indispensable^4,5^. However, the complex and variable nature of the marine environment presents a series of significant challenges for data collection and utilization. These include time-varying channel states, limited energy supplies, and additional constraints such as limited technological infrastructure^6^. Moreover, given the irreplaceable nature of marine data collection and utilization in fields such as climate change, biodiversity, and resource development^7^, it is crucial to investigate low-carbon and efficient data collection and utilization strategies for marine networks to support sustainable development goals^8,9^.

Currently, some researches focus on investigating the application of unmanned surface vehicles (USVs) in marine edge computing^10–13^. These vehicles aim to significantly improve the efficiency of data processing by transmitting substantial volumes of data collected by marine devices to edge servers^14–16^. However, they are often incapable of promptly addressing equipment service demands because of inherent limitations in navigation speed. In addition, dynamic and unpredictable fluctuations in the marine surface may disrupt the communication link, thereby affecting the stability of data transmission^17,18^.

The incorporation of non-ground-based computational nodes, such as unmanned aerial vehicles (UAVs)^19^, opens novel opportunities in marine communications. These computational nodes are unrestricted by terrain limitations and can be rapidly deployed to mission areas^20^, ensuring reliable communication support for the marine network from the air^21^. UAVs are not only capable of capturing and transmitting real-time marine surface images through the high-precision sensors they carry, which greatly enhances the timeliness and accuracy of marine monitoring^22,23^, but also act as a communication relay station to extend the data processing capacity of marine base stations. Supported by integrated sensing and communication technologies, UAVs can efficiently collect environmental data and instantly transmit information, offering robust technical assistance for the regulation and protection of marine resources^24–26^.

### Related works

In recent years, UAVs have been widely adopted for marine applications, primarily for data collection and communication relays^27–29^. In^30^, a sea-air integrated network model was proposed, wherein low-Earth orbit (LEO) satellites and UAVs jointly provide edge services to marine devices. The total energy consumption of UAVs with battery constraints was optimized by jointly managing communication resources, computational resources, and UAV path planning to enhance operational efficiency. Liu et al.^31^ investigated a marine mobile edge computing (MEC) system for efficient data sensing and introduced the concept of information age as a metric to evaluate data timeliness. They achieved a reduction in the average information age of Internet devices by jointly optimizing scheduling strategies for UAV trajectories, the total number of time slots, and data sensing. In^32^, Zhang et al. considered a scenario involving a fixed-wing UAV for data collection from buoys at sea. The study optimized communication time scheduling between buoys and the UAV’s wind-affected flight trajectory to minimize UAV energy consumption during the task. In^33^, a UAV-assisted marine monitoring network model was introduced, where a UAV hovers above an aggregation node and relays data from sunken nodes to a ground base station via a wireless communication link. Furthermore,^34^ and^35^ presented UAV-based solutions for efficient maritime wireless communication coverage. However, these methods rely on a single UAV to perform maritime tasks, which makes it difficult to ensure reliable service. For instance, excessive flight distances may lead to signal loss, and quickly retrieving drones in emergencies is difficult.

Owing to their unique capabilities, the collaboration between ships and UAVs for marine data collection has garnered significant interest from academia and industry alike. This collaboration can capitalize on the flexibility of UAVs and the robustness of ships for complex marine missions. In^36^, a study explored the collaboration of UAVs and ships for offloading decisions to support maritime missions. The ship was utilized for computation offloading in response to the limited endurance and computational capacity of UAVs. Specifically, in this study, the long-term constraints of total execution time and energy cost were transformed into short-term constraints using Lyapunov optimization. In^37^, Luo et al. introduced a hierarchical computation offloading framework for UAV-assisted marine communication networks based on game theory. They suggested offloading sensed information to USVs and UAVs to enhance task offloading efficiency. In^38^, a study examined a scenario where multiple hovering UAVs function as airborne edge servers to provide computational services. The study addressed scenarios involving malicious nodes eavesdropping on USV offloading transmissions and reduced overall energy consumption by optimizing the uploading time of underwater sensors, computation offloading by USVs, and confidentiality configurations. Although these studies demonstrate that collaboration between ships and UAVs can improve the efficiency of data processing and transmission and facilitate the development of adaptable and efficient communication networks in resource-constrained marine environments, significant challenges remain when UAVs are treated as edge servers to execute tasks. Specifically, UAVs have limited processing capabilities, and the additional computational load increases energy consumption. Moreover, the difficulty of recharging and dissipating heat in maritime settings may further compromise their performance and reliability.

Unlike the above studies, we propose a novel collaborative model that integrates UAVs and ships, focusing on a dual-UAV-assisted data relay collection system to minimize the total energy consumption of marine networks. Instead of treating UAVs as edge servers, we define them as data relay nodes responsible solely for data transmission without executing tasks, significantly reducing the payload on UAVs and making data collection more efficient and reliable.

**Table 1:** Summary of key notations.

Notation	Definition
$\mathcal {U}=\{1,2\}$	The set of UAVs
$\mathcal {M}=\{1,2,...,M\}$	The set of buoys
*T*	The service period
$\mathcal {N}=\{1,2,...,N\}$	The set of time slot
$\delta _t$	The duration of time slot
$q_{m}^{mu}=(x_{m}^{mu},y_{m}^{mu},0)$	The coordinates of buoy $m\in \mathcal {M}$
$q_{u}^{uav}[n]=(x_{u}^{uav}[n],y_{u}^{uav}[n],H)$	The coordinates of UAV $u\in \mathcal {U}$ in time slot $n\in \mathcal {N}$
$q^{ship}[n]=(x^{ship}[n],y^{ship}[n],0)$	The coordinates of the ship in time slot $n\in \mathcal {N}$
$B_{mu}^{uav}$/$B_{uav}^{ship}$/$B_{mu}^{ship}$	The wireless channel bandwidths between the buoys and the UAVs / the UAVs and the ship / the buoys and the ship
$h_{m}^{u}[n]$/$h_{u}^{ship}[n]$/$h_{m}^{ship}[n]$	The wireless channel gains between the buoys and the UAVs / the UAVs and the ship / the buoys and the ship
$\beta _0$	The channel gain per unit reference distance
$r_{m}^{u}[n]$/$r_{m}^{ship}[n]$/$r_{u}^{ship}[n]$	The transmission rate between buoy $m\in \mathcal {M}$ and UAV $u\in \mathcal {U}$ / buoy $m\in \mathcal {M}$ and the ship / UAV $u\in \mathcal {U}$ and the ship
$p_m[n]$	The transmit power of buoy $m\in \mathcal {M}$ in time slot $n\in \mathcal {N}$
$\sigma ^2$	The additive Gaussian white noise
$p_u[n]$	The transmit power of UAV $u\in \mathcal {U}$ in time slot $n\in \mathcal {N}$
*Q*	The minimum transmission rate between the UAVs and the ship
$L_m$	The amount of data collected by buoy $m\in \mathcal {M}$
$\varvec{\alpha }_{m}^{u}=\left\{ \alpha _{m}^{u}[ n \right] |m\in \mathcal {M},u\in \mathcal {U},n\in \mathcal {N} \}$	The access relationship between the buoys and the UAVs in each time slot
$\varvec{\alpha }_{m}^{ship}=\{ \alpha _{m}^{ship}[n] |m\in \mathcal {M} ,n\in \mathcal {N} \}$	The access relationship between the buoys and the ship in each time slot
$l_m[n]$	The amount of data offloaded from buoy $m\in \mathcal {M}$ in time slot $n\in \mathcal {N}$
$T_m^u[n]$/$T_m^{ship}[n]$/$T_u^{ship}[n]$	The delay incurred by transmitting the data from buoy $m\in \mathcal {M}$ to UAV $u\in \mathcal {U}$ / buoy $m\in \mathcal {M}$ to the ship / UAV $u\in \mathcal {U}$ to the ship in time slot $n\in \mathcal {N}$
$E_m^u$/$E_m^{ship}$/$E_u^{ship}$	The energy consumption incurred by transmitting the data from buoy $m\in \mathcal {M}$ to UAV $u\in \mathcal {U}$ / buoy $m\in \mathcal {M}$ to the ship / UAV $u\in \mathcal {U}$ to the ship in time slot $n\in \mathcal {N}$
$E_{tot}$	The total system energy consumption

### Motivation and contributions

Although UAVs have demonstrated great potential in the marine environment, significant challenges persist in efficiently utilizing their resources. A major challenge lies in ensuring UAVs can effectively perform their data-collection tasks while meeting low-carbon requirements. In this paper, we conduct an in-depth analysis of cooperative mechanisms between UAVs and ships in the marine environment. Furthermore, we propose a dual-UAV-assisted relay data collection model for marine networks and develop algorithms to enhance network resource efficiency. The major contributions of this work are summarized as follows.

1. We formulate the joint optimization problem of access relationship, UAV trajectory, data offloading, and communication resource allocation, aiming to minimize the total system energy consumption of the dual-UAV-assisted relay maritime data collection network system.
2. We decompose the joint optimization problem into four sub-problems by the block coordinate descent (BCD), i.e., access relationship, UAV trajectory, data offloading, and communication resource allocation. We devise an alternating optimization algorithm to obtain the near-optimal solution by solving the four sub-prob-lems alternately.
3. Extensive simulations are performed to demonstrate the effectiveness of the proposed algorithm under different scenarios. The numerical results show that the proposed algorithm outperforms other benchmark algorithms in terms of total system energy consumption.

### Organization

The rest of the paper is organized as follows. Section System model and problem formulation introduces the system model and problem formulation for the dual-UAV-assisted relay data collection problem. The details of the proposed algorithm are then presented in Section Approaches. The simulation and numerical results are provided to evaluate the performance of the proposed algorithm in Section Experimental analysis, followed by the conclusions in Section Conclusions.

## System model and problem formulation

**Fig. 1:**
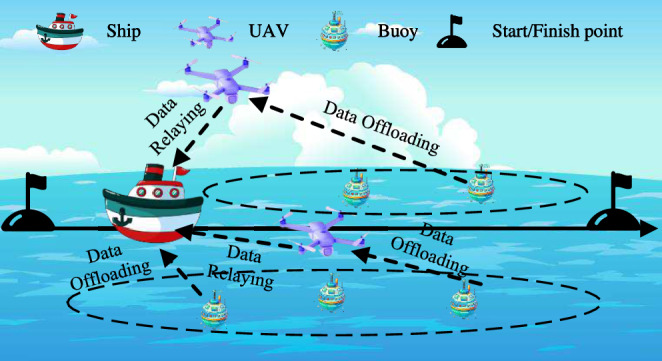
The dual-UAV-assisted relay maritime data collection network system.

As shown in Fig. 1, we consider a dual-UAV-assisted relay maritime data collection network system. The network consists of two rotary-wing UAVs for data relay, a ship that travels at a constant speed from start to finish and is capable of providing edge computing services, and *M* buoys for maritime data collection. Denote the set of UAVs and buoys as $\mathcal {U}=\{1,2\}$ and $\mathcal {M}=\{1,2,...,M\}$. For the considered service period *T*, it is divided into *N* time slots of equal duration $\delta _t$, and their set is represented as $\mathcal {N}=\{1,2,...,N\}$. A three-dimensional Cartesian coordinate system is employed to represent the coordinates of buoys, UAVs, and ship. We assume that the position of the buys in *T* is fixed, the coordinates of buoy $m\in \mathcal {M}$ can be represented by $q_{m}^{mu}=(x_{m}^{mu},y_{m}^{mu},0)$. Similarly, $q_{u}^{uav}[n]=(x_{u}^{uav}[n],y_{u}^{uav}[n],H)$ and $q^{ship}[n]=(x^{ship}[n],y^{ship}[n],0)$ are denoted as the coordinates of UAV $u\in \mathcal {U}$ and the ship in time slot $n\in \mathcal {N}$, respectively, where *H* is the flight altitude of UAVs. Since the ship is specifically designed for data collection and executing tasks, its initial trajectory can be designed to ensure that the buoys are as evenly distributed as possible on both sides. For ease of reference, the key notations used throughout this paper are listed in Table 1.

### Communication model

We represent the wireless channel bandwidths between the buoys and the UAVs, the UAVs and the ship, and the buoys and the ship by $B_{mu}^{uav}$, $B_{uav}^{ship}$, and $B_{mu}^{ship}$, respectively. Meanwhile, to guarantee the stability of maritime communication, the ship carries multiple antennas, which can communicate with multiple wireless nodes at the same time without interference. For reflecting the channel environment closer to the reality, in the time slot, the free space path loss model is used to construct the wireless channel models from buoys to UAVs, from UAVs to ship, and from buoys to ship, so that their wireless channel gains can be respectively denoted as

1 $$\begin{aligned} \begin{aligned} h_{m}^{u}[n] =&\frac{\beta _0}{\left\| q_{u}^{uav}\left[ n \right] -q_{m}^{mu}[ n \right] \Vert ^2},\\ &\ \ \ \ \ \ \ \ \ \ \ \ \ \ \ \ \ \ \ \ \ \ \forall m\in \mathcal {M} ,u\in \mathcal {U} ,n\in \mathcal {N} , \end{aligned} \end{aligned}$$

2 $$\begin{aligned} h_{u}^{ship}[n] =\frac{\beta _0}{\left\| q^{ship}\left[ n \right] -q_{u}^{uav}[n] \right\| ^2},\forall u\in \mathcal {U} ,n\in \mathcal {N} , \end{aligned}$$

3 $$\begin{aligned} h_{m}^{ship}[n] =\frac{\beta _0}{\left\| q^{ship}\left[ n \right] -q_{m}^{mu}[n] \right\| ^2},\forall m\in \mathcal {M} ,n\in \mathcal {N} , \end{aligned}$$

where $\beta _0$ is the channel gain per unit reference distance, and $\left\| \cdot \right\|$ is the Euclidean norm.

For ensuring the integrity and continuity of data transmission, it is assumed that UAV $u\in \mathcal {U}$ provides data relay service for only $C_u$ buoys in *T*. The set of these buoys is denoted by $\mathcal {M}_u$. In addition, to avoid multi-access interference caused by simultaneous data transmission from multiple buoys, time division multiple access (TDMA) is adopted to improve the stability and quality of the wireless channel^39^. In time slot $n\in \mathcal {N}$, the transmission rate between buoy $m\in \mathcal {M}$ and UAV $u\in \mathcal {U}$ can be expressed as

4 $$\begin{aligned} \begin{aligned} r_{m}^{u}[ n ] =&B_{mu}^{uav}\log _2\left( 1+\frac{p_m[ n ] h_{m}^{u}[ n ]}{\sigma ^2} \right) ,\\&\ \ \ \ \ \ \ \ \ \ \ \ \ \ \ \ \ \ \ \ \ \forall m\in \mathcal {M} ,u\in \mathcal {U} ,n\in \mathcal {N} , \end{aligned} \end{aligned}$$

where $p_m[n]$ is the transmit power of buoy $m\in \mathcal {M}$ in time slot $n\in \mathcal {N}$, and $\sigma ^2$ is the additive Gaussian white noise.

The ship in the network carries multiple antennas for wireless communication with the buoys. Considering the long distance between buoys at sea, it is assumed that the ship will not interfere with each other when communicating with several buoys at the same time. In time slot $n\in \mathcal {N}$, the transmission rate between buoy $m\in \mathcal {M}$ and the ship can be expressed as

5 $$\begin{aligned} \begin{aligned} r_{m}^{ship}[ n ] =&B_{mu}^{ship}\log _2\left( 1+\frac{p_m[ n ] h_{m}^{ship}\left[ n \right] }{\sigma ^2} \right) ,\\ &\ \ \ \ \ \ \ \ \ \ \ \ \ \ \ \ \ \ \ \ \ \ \ \ \ \ \forall m\in \mathcal {M} ,n\in \mathcal {N} , \end{aligned} \end{aligned}$$

Furthermore, the wireless communication between the ship and the UAVs in the network is carried out using frequency division multiple access (FDMA). In time slot $n\in \mathcal {N}$, the transmission rate between UAV $u\in \mathcal {U}$ and the ship can be expressed as

6 $$\begin{aligned} \begin{aligned} r_{u}^{ship}[n] =&\frac{B_{uav}^{ship}}{U}\log _2\left( 1+\frac{p_u[n] h_{u}^{ship}[n]}{\sigma ^2} \right) ,\\ &\ \ \ \ \ \ \ \ \ \ \ \ \ \ \ \ \ \ \ \ \ \ \ \ \ \ \ \ \forall u\in \mathcal {U} ,n\in \mathcal {N} , \end{aligned} \end{aligned}$$

where $p_u[n]$ is the transmit power of UAV $u\in \mathcal {U}$ in time slot $n\in \mathcal {N}$. To guarantee the reliability and stability of the communication transmission between the UAVs and the ship, the minimum transmission rate between them is required to be *Q*, i.e.,

7 $$\begin{aligned} r_{u}^{ship}[n] \ge Q,\forall u\in \mathcal {U} ,n\in \mathcal {N} . \end{aligned}$$

### Transmission, relay, and offloading model

**Fig. 2:**
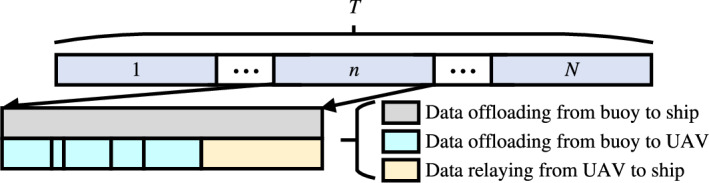
The diagram of time slot.

As shown in Fig. 2, there are two simultaneous processes in *T*, i.e., the data from the buoy are offloaded directly to the ship and the data from the buoy are transmitted to the UAV and then relayed to the ship. It should be noted that both processes are constrained by the duration of the time slot.

Let $L_m$ denote the amount of data collected by buoy $m\in \mathcal {M}$ prior to the start of the service period, which will be relayed by UAV or offloaded directly to the ship within *T*. In view of the impact of the UAV’s load on the flight, the UAV is only used as a relay device, which can greatly reduce the overall quality of the UAV and ensure a longer endurance. The edge servers on board the ship are utilized to handle the collected data. To improve the utilization of communication resources in the network system, we adopt a partial offloading strategy. Specifically, the buoy can split the data collected into two parts, one relayed to the ship via a UAV and the other offloaded directly to the ship. We define a binary variable $\varvec{\alpha }_{m}^{u}=\left\{ \alpha _{m}^{u}[ n \right] |m\in \mathcal {M},u\in \mathcal {U},n\in \mathcal {N} \}$ to represent the access relationship between the buoys and the UAVs, when buoy $m\in \mathcal {M}$ relays part of the data to the ship via UAV $u\in \mathcal {U}$ in time slot $n\in \mathcal {N}$, $\alpha _{m}^{u}[n] =1$, otherwise $\alpha _{m}^{u}[n] =0$. Similarly, let $\varvec{\alpha }_{m}^{ship}=\{ \alpha _{m}^{ship}[n] |m\in \mathcal {M},n\in \mathcal {N} \}$ denote the access relationship between the buoys and the ship in each time slot, when buoy $m\in \mathcal {M}$ directly offloads part of the data to the ship in time slot $n\in \mathcal {N}$, $\alpha _{m}^{ship}[n] =1$, otherwise $\alpha _{m}^{ship}[n] =0$. We assume that data division does not generate additional data volume, this setting has been widely used^40^. Specifically, each buoy can communicate with only one device (UAV or ship) in each time slot, i.e.,

8 $$\begin{aligned} \sum _{u=1}^U{\alpha _{m}^{u}[ n ]}+\alpha _{m}^{ship}[ n ] \le 1,\forall m\in \mathcal {M} ,n\in \mathcal {N} . \end{aligned}$$

Furthermore, we indicate the amount of data offloaded from buoy $m\in \mathcal {M}$ in time slot $n\in \mathcal {N}$ by $l_m[n]$, and the delay and energy consumption incurred by transmitting these data to UAV $u\in \mathcal {U}$ can be denoted as

9 $$\begin{aligned} T_{m}^{u}[ n ] =\frac{\alpha _{m}^{u}[ n ] l_m[ n ]}{r_{m}^{u}[ n ]},\forall n\in \mathcal {N} ,u\in \mathcal {U} ,m\in \mathcal {M} , \end{aligned}$$

10 $$\begin{aligned} E_{m}^{u}=\sum _{n=1}^N{T_{m}^{u}[ n ] p_m[ n ]},\forall u\in \mathcal {U} ,m\in \mathcal {M} . \end{aligned}$$

Similarly, the delay and energy consumption incurred by buoy $m\in \mathcal {M}$ to offload data to the ship in time slot $n\in \mathcal {N}$ can be expressed as

11 $$\begin{aligned} T_{m}^{ship}[ n ] =\frac{\alpha _{m}^{ship}[ n ] l_m[ n ]}{r_{m}^{ship}[ n ]},\forall n\in \mathcal {N} ,m\in \mathcal {M} , \end{aligned}$$

12 $$\begin{aligned} E_{m}^{ship}=\sum _{n=1}^N{T_{m}^{ship}[ n ] p_m[ n ]},\forall m\in \mathcal {M} . \end{aligned}$$

Further, the delay and energy consumption incurred by UAV $u\in \mathcal {U}$ to relay data to the ship in time slot $n\in \mathcal {N}$ can be expressed as

13 $$\begin{aligned} T_{u}^{ship}[n] =\frac{\sum _{m=1}^M{\alpha _{m}^{u}[n] l_m[ n ]}}{r_{u}^{ship}[ n ]},\forall n\in \mathcal {N} ,u\in \mathcal {U} , \end{aligned}$$

14 $$\begin{aligned} E_{u}^{ship}=\sum _{n=1}^N{T_{u}^{ship}\left[ n \right] p_u\left[ n \right] },\forall u\in \mathcal {U} . \end{aligned}$$

In summary, the total system energy consumption for all data collected by the buoys to be offloaded to the ship is

15 $$\begin{aligned} E_{tot}=\sum _{u=1}^U{E_{u}^{ship}}+\sum _{m=1}^M{E_{m}^{ship}}+\sum _{u=1}^U{\sum _{m=1}^M{E_{m}^{u}}}. \end{aligned}$$

### Data collection model

In marine application scenarios, the buoys usually lack computational capabilities and their main function is to detect, collect, and store data. To ensure that complete data can be collected, the amount of data offloaded by the buoy in a period should be no less than the amount of collected data by the buoy, i.e.,

16 $$\begin{aligned} \sum _{n=1}^N{l_m\left[ n \right] \ge L_m,}\forall m\in \mathcal {M} . \end{aligned}$$

### Problem formulation

In this paper, we are dedicated to jointly optimizing access relationship $\varvec{\alpha }=\{\alpha _m^u[n],\alpha _m^{ship}[n]|m\in \mathcal {M},u\in \mathcal {U},n\in \mathcal {N}\}$, UAV trajectory $\varvec{q}=\{q_u^{uav}[n]|u\in \mathcal {U},n\in \mathcal {N}\}$, data offloading $\varvec{l}=\{l_m[n]|m\in \mathcal {M},n\in \mathcal {N}\}$, and communication resource allocation $\varvec{p}=\{p_u[n]|u\in \mathcal {U},n\in \mathcal {N}\}$ to minimize the total system energy consumption. We define the formulated problem as

17 $$\begin{aligned}&P:\mathop {\min }\limits _{\varvec{\alpha },\varvec{q},\varvec{l}, \varvec{p}} \quad E_{tot}&\nonumber \\ \text{ s.t. }\quad \end{aligned}$$

C1 $$\begin{aligned}&\sum \nolimits _{u=1}^U{\alpha _{m}^{u}[n] +\alpha _{m}^{ship}[ n ]}\le 1,\forall m,n&\end{aligned}$$

C2 $$\begin{aligned}&\alpha _{m}^{u}[n] ,\alpha _{m}^{ship}[n] \in \{ 0,1 \} ,\forall m,u,u&\end{aligned}$$

C3 $$\begin{aligned}&\alpha _{m}^{ship}[n] d_{m}^{ship}[n] \le R_{ship}^{\max },\forall m,n&\end{aligned}$$

C4 $$\begin{aligned}&0\le p_u[n] \le P_{u}^{\max },\forall u,n&\end{aligned}$$

C5 $$\begin{aligned}&q_{u}^{uav}[1] =q^{ship}[1],q_{u}^{uav}[N] =q^{ship}[N] ,\forall u&\end{aligned}$$

C6 $$\begin{aligned}&0\le T_{m}^{u}[n] \le \delta _t,\forall m,u,u&\end{aligned}$$

C7 $$\begin{aligned}&0\le T_{m}^{ship}[n] \le \delta _t,\forall m,n&\end{aligned}$$

C8 $$\begin{aligned}&0\le T_{u}^{ship}[n] \le \delta _t,\forall u,u&\end{aligned}$$

C9 $$\begin{aligned}&T_{u}^{ship}[n] +\sum \nolimits _{m=1}^M{ T_{m}^{u}[n] }\le \delta _t,\forall u,n&\end{aligned}$$

C10 $$\begin{aligned}&\sum \nolimits _{n=1}^N{l_m[ n ]}\ge L_m,\forall m&\end{aligned}$$

C11 $$\begin{aligned}&(\left\| q_{u}^{uav}\left[ n+1 \right] -q_{u}^{uav}[ n ] \right\| ) / \delta _t\le V_{uav}^{\max },\forall u,n&\end{aligned}$$

C12 $$\begin{aligned}&r_{u}^{ship}\left[ n \right] \ge Q,\forall u,n&\end{aligned}$$

C1 denotes the access relationship constraints between buoys, UAVs, and ship. C2 is a value constraint on the optimization variable $\varvec{\alpha }$. C3 indicates that the buoy needs to be within the service range of the ship to be accessed. C4 is the constraint on the value of the optimization variable $\varvec{p}$, where $P_{u}^{\max }$ indicates the maximum value of the transmit power for UAV $u\in \mathcal {U}$. C5 represents the drones to take off from and eventually land on the ship. C6-C9 represent delay constraints. C10 means that all data from the buoy is to be transmitted to the ship. C11 indicates the flight constraint, where $V_{uav}^{\max }$ is the maximum flight speed of the UAVs. C12 is the minimum transmission rate constraint between the UAVs and the ship.

## Approaches

To facilitate the collection of data from each buoy, we partitioned *M* buoys into two groups according to the left and right sides of the ship’s navigation trajectory, with one UAV assigned to each group for data relay and offloading. Given that *P* is formulated as a complex nonlinear and non-convex integer optimization problem, we resolved variable coupling by employing a combination of a greedy algorithm, BCD, and sequential convex approximation (SCA). This approach enabled the decomposition of the problem into two distinct parts. The first part applies a greedy algorithm to resolve the access relationship sub-problem ($P_1$). The second part employs BCD to further partition the remaining problem into specific sub-problems:

1. UAV trajectory optimization ($P_2$): The UAV trajectory $\varvec{q}$ is optimized using the same given data offloading $\varvec{l}$ and communication resource allocation $\varvec{p}$, along with the optimized access relationship $\varvec{\alpha }$ obtained in the first sub-problem; (Section UAV trajectory optimization with fixed access relationship, data offloading, and communication resource allocation)
2. Data offloading optimization ($P_3$): The data offloading $\varvec{l}$ is optimized by solving the problem using the given communication resource allocation $\varvec{p}$, as well as the optimized access relationship $\varvec{\alpha }$ and UAV trajectory $\varvec{q}$. (Section Data offloading optimization with fixed access relationship, UAV trajectory, and communication resource allocation).
3. Communication resource allocation optimization ($P_4$): The communication resource allocation $\varvec{p}$ is optimized by solving the problem using the previously determined access relationship $\varvec{\alpha }$, UAV trajectory $\varvec{q}$, and data offloading $\varvec{l}$. (Section Communication resource allocation optimization with fixed access relationship, UAVtrajectory, and data offloading)

An approximate optimal solution to *P* is obtained through iterative resolution of its four sub-problems.

### Access relationship optimization with fixed UAV trajectory, data offloading, and communication resource allocation

Optimizing the access relationship $\varvec{\alpha }$ is accomplished using the given UAV trajectory $\varvec{q}$, data offloading $\varvec{l}$, and communication resource allocation $\varvec{p}$. Using these parameters as inputs, we can express the sub-problem $P_1$ as

18 $$\begin{aligned}&P_1:\mathop {\min }\limits _{\varvec{\alpha }} \quad E_{tot}&\nonumber \\ \text{ s.t. }\quad&C1-C3, C6-C9 \end{aligned}$$

Given that the optimization variables are binary, $P_1$ is classified as an integer programming problem. A greedy algorithm is utilized to address this problem. Assuming that UAVs provide aerial relay services and the maritime environment lacks signal-blocking obstacles, the service coverage of the UAVs is considered to cover the entire area, ensuring continuous buoy connectivity to their corresponding UAVs. The greedy algorithm ensures that buoys within the ship’s service coverage directly access the ship without relying on UAV relay services. The details of the access relationship optimization algorithm are described in Algorithm 1.

**Algorithm 1:**
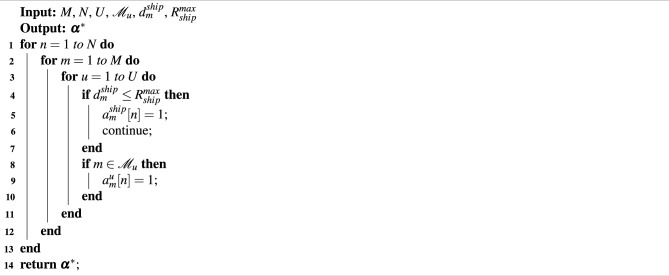
Access relationship optimization based on Greedy Algorithm.

### UAV trajectory optimization with fixed access relationship, data offloading, and communication resource allocation

Optimizing the UAV trajectory $\varvec{q}$ is accomplished using the given data offloading $\varvec{l}$, communication resource allocation $\varvec{p}$ and the access relationship $\varvec{\alpha }^*$ established as previously discussed. Using these parameters as inputs, we can express the sub-problem $P_2$ as

19 $$\begin{aligned}&P_2:\mathop {\min }\limits _{\varvec{q}} \quad E_{tot}&\nonumber \\ \text{ s.t. }\quad&C5, C6, C8, C9, C11, C12 \end{aligned}$$

Constraints C6, C8, C9, and C12 are non-convex with respect to $\varvec{q}$, rendering sub-problem $P_2$ non-convex. Here, we transform $P_2$ into a convex optimization problem by employing successive convex approximation. Initially, the transmission rate between the buoys and the UAV is re-expressed as

20 $$\begin{aligned} \begin{aligned} r_{m}^{u}[ n ]=&B_{mu}^{uav}\log _2\left( \frac{p_m[ n ] \beta _0}{\Vert q_{u}^{uav}[ n ] -q_{m}^{mu}[ n ] \Vert ^2}+\sigma ^2 \right) \\ &-B_{mu}^{uav}\log _2( \sigma ^2 ) \\ =&\hat{r}_{m,u}[ n ] -B_{mu}^{uav}\log _2( \sigma ^2 ) . \end{aligned} \end{aligned}$$

In accordance with the SCA method, the global lower bound function of $\hat{r}_{m,u}[n]$ can be substituted with its first-order Taylor expansion at the j-th iteration for any specified local point $q_{u}^{j}[n]$. Similarly, $r_{m}^{u}[n]$ can be approximated by its lower bound function, i.e.,

21 $$\begin{aligned} r_{m}^{u}[ n ] \ge \hat{r}_{m}^{u}[ n ] =r_{m,u}^{lb}[ n ] -B_{mu}^{uav}\log _2( \sigma ^2 ) . \end{aligned}$$

Among them, $\hat{r}_{m}^{u}[n]$ represents the lower bound function of $r_{m}^{u}[n]$, and $r_{m,u}^{lb}[n]$ is the lower bound function of $\hat{r}_{m,u}[n]$. We can see from Eq. (22) that, in $r_{m,u}^{lb}[n]$, $\Vert q_{u}^{uav}[n] -q_{u}^{j}[n] \Vert$ is convex with respect to $q_{u}^{uav}[n]$.

22 $$\begin{aligned} \begin{aligned} \hat{r}_{m,u}\left[ n \right] =&B_{mu}^{uav}\log _2\left( \frac{p_m\left[ n \right] \beta _0}{\Vert q_{u}^{uav}\left[ n \right] -q_{m}^{mu}\left[ n \right] \Vert ^2}+\sigma ^2 \right) \\ \ge&B_{mu}^{uav}\log _2\left( \frac{p_m\left[ n \right] \beta _0}{\Vert q_{u}^{j}\left[ n \right] -q_{m}^{mu}\left[ n \right] \Vert ^2}+\sigma ^2 \right) -B_{mu}^{uav}\left( \frac{2\frac{p_m\left[ n \right] \beta _0\left( \Vert q_{u}^{j}\left[ n \right] -q_{m}^{mu}\left[ n \right] \Vert \right) \left( \Vert q_{u}^{uav}\left[ n \right] -q_{u}^{j}\left[ n \right] \Vert \right) }{\left( \Vert q_{u}^{j}\left[ n \right] -q_{m}^{mu}\left[ n \right] \Vert ^2 \right) ^2}}{\ln 2\left( \frac{p_m\left[ n \right] \beta _0}{\Vert q_{u}^{j}\left[ n \right] -q_{m}^{mu}\left[ n \right] \Vert ^2}+\sigma ^2 \right) } \right) =r_{m,u}^{lb}\left[ n \right] , \end{aligned} \end{aligned}$$

Similarly, the transmission rate between UAV and ship can be obtained through the aforementioned steps by employing Eq. (23) for the lower bound function. $\hat{r}_{u}^{ship}[n]$ represents the lower bound function of $r_{u}^{ship}[n]$, and $\Vert q_{u}^{uav}[n] -q_{u}^{j}[n] \Vert$ is convex relative to $q_{u}^{uav}[n]$.

23 $$\begin{aligned} \begin{aligned} r_{u}^{ship}\left[ n \right] =&\left( B_{uav}^{ship}/U \right) \log _2\left( \frac{p_u\left[ n \right] \beta _0}{\Vert q^{ship}\left[ n \right] -q_{u}^{uav}\left[ n \right] \Vert ^2}+\sigma ^2 \right) -\left( B_{uav}^{ship}/U \right) \log _2\left( \sigma ^2 \right) \\ \ge&\left( B_{uav}^{ship}/U \right) \log _2\left( \frac{p_u\left[ n \right] \beta _0}{\Vert q^{ship}\left[ n \right] -q_{u}^{j}\left[ n \right] \Vert ^2}+\sigma ^2 \right) -\left( B_{uav}^{ship}/U \right) \log _2\left( \sigma ^2 \right) \\ &-\left( B_{uav}^{ship}/U \right) \left( \frac{\frac{p_u\left[ n \right] \beta _0\left( \Vert q^{ship}\left[ n \right] -q_{u}^{uav}\left[ n \right] \Vert ^2-\Vert q^{ship}\left[ n \right] -q_{u}^{j}\left[ n \right] \Vert ^2 \right) }{\left( \Vert q^{ship}\left[ n \right] -q_{u}^{j}\left[ n \right] \Vert ^2 \right) ^2}}{\ln 2\left( \frac{p_m\left[ n \right] \beta _0}{\Vert q^{ship}\left[ n \right] -q_{u}^{j}\left[ n \right] \Vert ^2}+\sigma ^2 \right) } \right) \ =\hat{r}_{u}^{ship}\left[ n \right] , \end{aligned} \end{aligned}$$

It is now necessary to reinsert $\hat{r}_{m}^{u}[n]$ and $\hat{r}_{u}^{ship}[n]$ into $T_{m}^{u}$ and $T_{u}^{ship}$ and to reformulate constraints C6 and C8 as ĉ6 and ĉ8. Consequently, by replacing the derived lower bound function and the approximate convex expression, the objective function of the sub optimization problem for UAV trajectory design $\varvec{q}$ is reformulated as

24 $$\begin{aligned} \begin{aligned} \hat{E}_{tot}=&\sum _{u=1}^U{\sum _{n=1}^N{\frac{\sum \limits _{m=1}^M{\alpha _{m}^{u}\left[ n \right] l_m\left[ n \right] }}{\hat{r}_{u}^{ship}\left[ n \right] }p_u\left[ n \right] }}+\sum _{m=1}^M{E_{m}^{ship}}\\ &+\sum _{u=1}^U{\sum _{m=1}^M{\sum _{n=1}^N{\frac{\alpha _{m}^{u}\left[ n \right] l_m\left[ n \right] }{\hat{r}_{m}^{u}\left[ n \right] }p_m\left[ n \right] }}}. \end{aligned} \end{aligned}$$

In conclusion, $P_2$ can be rewritten as follows:

25 $${P_{{2.1}} :\mathop {\min }\limits_{q} \quad \hat{E}_{{tot}} }$$

C6 $${{\text{s}}{\text{.t}}{\text{.}}\quad } \quad {\frac{{\alpha _{m}^{u} \left[ n \right]l_{m} \left[ n \right]}}{{\hat{r}_{m}^{u} \left[ n \right]}} \le \delta _{t} ,\forall m,u,u}$$

C8 $${\frac{{\sum\limits_{{m = 1}}^{M} {\alpha _{m}^{u} \left[ n \right]l_{m} \left[ n \right]} }}{{\hat{r}_{u}^{{ship}} \left[ n \right]}} \le \delta _{t} ,\forall u,u}$$

C9 $${\frac{{\sum\limits_{{m = 1}}^{M} {\alpha _{m}^{u} \left[ n \right]l_{m} \left[ n \right]} }}{{\hat{r}_{u}^{{ship}} \left[ n \right]}} + \sum\limits_{{m = 1}}^{M} {\left( {\frac{{\alpha _{m}^{u} \left[ n \right]l_{m} \left[ n \right]}}{{\hat{r}_{m}^{u} \left[ n \right]}}} \right)} \le \delta _{t} ,\forall u,n}$$

C12 $$\begin{gathered} \hat{r}_{u}^{{ship}} \left[ n \right] \ge Q,\forall u,n \hfill \\ {\text{C5}},{\text{ C11}} \hfill \\ \end{gathered}$$

$P_{2.1}$ is a standard convex optimization problem that can be solved using the CVX toolbox^41^.

### Data offloading optimization with fixed access relationship, UAV trajectory, and communication resource allocation

Optimizing the data offloading $\varvec{l}$ is accomplished using the given communication resource allocation $\varvec{p}$ as well as the access relationship $\varvec{\alpha }^*$ and UAV trajectory $\varvec{q}^*$ obtained above. Using these parameters as inputs, we can express the sub-problem $P_3$ as

26 $$\begin{aligned}&P_3:\mathop {\min }\limits _{\varvec{l}} \quad E_{tot}&\nonumber \\ \text{ s.t. }\quad&C6-C10 \end{aligned}$$

It is evident that sub-problem $P_3$ is a standard linear programming problem, given that the constraints are all linear constraints on data offloading $\varvec{l}$. Consequently, the optimal solution for data offloading $\varvec{l}$ can be readily obtained through the utilization of a convex optimization toolbox.

### Communication resource allocation optimization with fixed access relationship, UAV trajectory, and data offloading

Optimizing the communication resource allocation $\varvec{p}$ is achieved by utilizing the previously determined access relationship $\varvec{\alpha }^*$, UAV trajectory $\varvec{q}^*$ and data offloading $\varvec{l}^*$. Using these parameters as inputs, we can express the sub-problem $P_4$ as

27 $$\begin{aligned}&P_4:\mathop {\min }\limits _{\varvec{p}} \quad E_{tot}&\nonumber \\ \text{ s.t. }\quad&C4, C8, C9, C12 \end{aligned}$$

In the objective function, we consider the energy consumption, denoted as $E_{u}^{ship}$, which is associated with the communication transmission between the UAV and the ship. This energy consumption is constituted of the UAV transmission power $p_u$ and transmission delay $T_{u}^{ship}$, where $T_{u}^{ship}$ is also a function of $p_u$. It would be inadvisable to attempt to solve for $E_{u}^{ship}$ directly. To address this, we employ the relaxation function method to tackle the optimization problem. Based on the above sub-problems, the transmission delay between the buoy and the drone was obtained, and $\hat{T}_{u}^{ship}$ was used to represent the communication delay between the new drone and the ship. Among the aforementioned variables, $\hat{T}_{u}^{ship}$ represents the upper bound of $T_{u}^{ship}$:

28 $$\begin{aligned} \hat{T}_{u}^{ship}\left[ n \right] =\delta _t-\sum _{m=1}^M{\left( T_{m}^{u}\left[ n \right] \right) }. \end{aligned}$$

The objective function $E_{tot}\le \hat{E}_{tot}$, where

29 $$\begin{aligned} \hat{E}_{tot}=\sum _{u=1}^U{\sum _{n=1}^N{\hat{T}_{u}^{ship}\left[ n \right] p_u\left[ n \right] }}+\sum _{m=1}^M{E_{m}^{ship}}+\sum _{u=1}^U{\sum _{m=1}^M{E_{m}^{u}}}. \end{aligned}$$

The sub-problem $P_4$ can be rewritten as:

30 $$\begin{aligned}&P_{4.1}:\mathop {\min }\limits _{\varvec{p}} \quad \hat{E}_{tot}&\nonumber \\ \text{ s.t. }\quad&C4, C8, C9, C12 \end{aligned}$$

Secondly, it must be demonstrated that the constraints of $P_4$ are convex. C4 and C12 are linear constraints on $p_u$. The $T_{u}^{ship}$ in C8 and C9 is a function of $p_u$. Equivalent $T_{u}^{ship}$ as function $f(x) =1/\log ( 1+x)$. If the second derivative $f''(x) >0$ holds for all *x* in the domain, then the function is convex.

31 $$\begin{aligned} f''\left( x \right) =\frac{\log \left( 1+x \right) +2}{\left( 1+x \right) ^2\left( \log \left( 1+x \right) \right) ^3}. \end{aligned}$$

In accordance with the derivation formula of $f''(x)$, within the domain, $f''(x) > 0$. Consequently, constraints C4 and C8 are convex constraints, and problem $P_{4.1}$ can be solved using the CVX toolbox.

### Initialize the settings

This section initializes four critical parameters: access relationship $\varvec{\alpha }$, UAV trajectory $\varvec{q}^{1}$, data offloading $\varvec{l}^{1}$, and communication resource allocation $\varvec{p}^1$. The initialization details for each parameter are as follows:

1. Access Relationship: The initialization ensures that all buoys maintain continuous access to their designated UAVs throughout each cycle, thereby achieving seamless communication coverage.
2. UAV Trajectory: Each UAV is initialized with N + 1 waypoints, including a key hovering point whose coordinates are set as the centroid of the buoys it serves. The UAV first flies at maximum speed to the hovering point and remains there for a calculated period. The hovering time is determined to ensure that, at the end of the trajectory, the UAV can return to the ship at maximum speed, completing the entire flight within the allowed duration.
3. Data Offloading: The objective is to transfer all data in a single cycle and distribute it equitably across time slots, thereby ensuring a balanced load for data transmission.
4. Communication Resource Allocation: The UAV is configured to transmit data at the maximum permitted power output for the duration of the transmission cycle.

### Global algorithm

In conclusion, the initial problem *P* can be optimized through joint access relationship $\varvec{\alpha }=\{ \alpha _{m}^{u}[n],\alpha _{m}^{ship}[n] \}$, UAV trajectory $\varvec{q}=\{q_{u}^{uav}[n]\}$, data offloading $\varvec{l}=\{l_m[n]\}$, and communication resource allocation $\varvec{p}=\{p_u[n]\}$. However, as the solution to sub-problem $P_{2.1}$ is only applicable to atomic problem $P_2$, it cannot be guaranteed that the convergence point represents the global optimal solution. Nevertheless, relevant research^40^ findings indicate that the convergence point of the BCD iteration is at least a local optimum.

**Algorithm 2:**
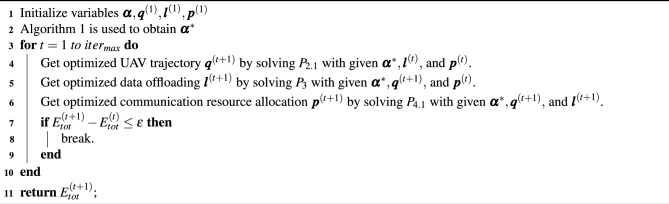
Total energy consumption minimization optimization algorithm based on BCD method.

**Table 2:** Simulation parameters.

Parameter	Value
*M*	10
*T*	{100, 150, 200} s
*N*	{100, 150, 200}
$B_{mu}^{uav}$	1 MHz
$B_{uav}^{ship}$	2 MHz
$B_{mu}^{ship}$	1 MHz
$\beta _0$	-37 dBm
*H*	100 m
$\sigma ^2$	-107 dBm
$R_{ship}^{max}$	{50, 100, 150} m$^2$
$V_{uav}^{max}$	25 m/s
$L_m$	{10, 20, 30, 40, 50, 60} MB
$p_m$	{0.4, 0.5, 0.6, 0.7, 0.8, 0.9, 1} W
$P_{u}^{max}$	1 W
*Q*	{1, 2, 3, 4} MBit/s

### Time complexity analysis

In solving $P_1$, the greedy algorithm we employ yields a near-optimal solution very quickly. Thus, the time complexity for solving $P_1$ is negligible. The software CVX, used for solving optimization problems, has a complexity of $O(\text {log}(1/\tilde{\omega })\tilde{n}^3)$^42^, where $\tilde{n}$ is the number of optimization variables and $\tilde{\omega }$ is the threshold^43^. For $P_2$, we apply a first-order Taylor expansion. Assuming *L*1 iterations are needed to reach the current approximate optimal solution, and considering the optimization variables include the *NUM* UAV trajectory variables $q^{uav}_u[n]$, the complexity becomes $O(L1*(\text {log}(1/\tilde{\omega })((U+1)*2*M)^3))$. Overall, the solution process of $P_3$ can be completed within a time complexity of $O(\text {log}(1/\tilde{\omega }(N*M)^3)$. When solving $P_4$, the time complexity is $O(\text {log}(1/\tilde{\omega })(N*U)^3)$.

Assuming that the algorithm requires *L* rounds of iterations to converge to an approximate optimal solution that satisfies the conditions, the complexity of the algorithm, according to the above analysis of the complexity of each subproblem solving process, is $O(L(L1*(\text {log}(1/\tilde{\omega })((U+1)*2*M)^3)) + \text {log}(1/\tilde{\omega })(N*M)^3 +\text {log}(1/\tilde{\omega })(N*U)^3)$.

## Discussion

### Wireless channel modeling considerations

We adopt the deterministic free-space path loss model, commonly used in prior studies^1,32,44^, as a reasonable approximation for open-sea communication. While this model omits fading, shadowing, and multipath effects, such simplification enables focused analysis on UAV trajectory design. In our future work, we will incorporate more realistic models, such as Rayleigh or Rician fading, to enhance environmental fidelity. Additionally, we assume interference-free FDMA/TDMA communication, similar to existing literature^1,32,44^. Although idealized, this is justified by the sparse communication environment in open seas. Prior maritime studies^45,46^ also indicate that interference is typically limited and manageable. Future research may consider interference-aware mechanisms to improve robustness.

### Spatiotemporal synchronization assumption

We assume perfect time and position synchronization among UAVs and ships, following the setting of existing studies^1,30,32^. While practical issues such as clock drift control latency, and navigation errors could compromise this assumption and impact system performance. Established techniques exist to minimize these errors to negligible levels. For instance, high-precision clock synchronization technologies, such as the IEEE 1588 Precision Time Protocol (PTP), can achieve nanosecond-level accuracy in distributed systems, ensuring temporal coordination between UAVs and ships. Additionally, modern navigation systems, such as Global Positioning System (GPS) and Inertial Navigation Systems (INS), provide high-accuracy positioning. Therefore, given that synchronization mechanisms are not the primary focus of this work, the idealized assumption is reasonable.

## Experimental analysis

### Simulation settings

We used a personal computer equipped with a 3.4GHz Intel Core i7 processor and 32GB of memory as the simulation platform and conducted numerical simulation experiments using Matlab. In the simulation, we consider a sea area of 800 m $\times$ 600 m, with 10 buoys randomly distributed within it. The ship travels maintains a constant speed from the starting point (0, 0) to the endpoint (800, 0). Table 2 presents the basic simulation parameters in the experiment^33,34,40^. In the absence of explicit system parameter specifications, the cycle length *T* is set to 100 seconds, the number of time slots *N* is 100, the maximum service coverage of the ship $R_{ship}^{max}$ is 50 meters, the data size collected by each buoy $L_m$ is 50 MB, the transmission power of each buoy $p_m$ is 0.5 W, and the minimum transmission rate between the UAV and the ship *Q* is 2 MBit/s. The maximum number of iterations of the system $iter_{\max }$ is set to 20, and the iteration accuracy $\epsilon$ is set to $10^{-5}$. The maximum number of iterations for the optimization of UAV trajectory coordinates $iter^{uav}_{\max }$ is 5.

### Benchmark algorithms

To better evaluate the performance of the proposed approach, we conduct the comparison by introducing benchmark algorithms^45^ as follows:

- (BAO) Buoys Access Only UAVs: This method ensures that all buoys have access to the appropriate UAVs in each time slot.
- (FTA) Fixed Trajectory Approach^47^: In this method, the trajectory of the UAV is predetermined, with the UAV following a fixed trajectory. All other variables are optimized using the methods described in this paper.
- (TPM) Transmission Power Maximization^48^: In this method, UAVs are configured to transmit data at their maximum transmission power at all times.
- (BAO+FTA+TPM=BFT) Data Offloading Allocation Optimization Only: This method integrates the aforementioned comparison methods, employing convex optimization techniques exclusively for the optimization of data offloading allocation.
- (ASF) UAV Accompanying Ship Flight: In this method, the trajectory of the UAV is set to be identical to that of the ship, while all other variables are optimized using the methods described in this paper.

### Overall performance analysis

**Fig. 3:**
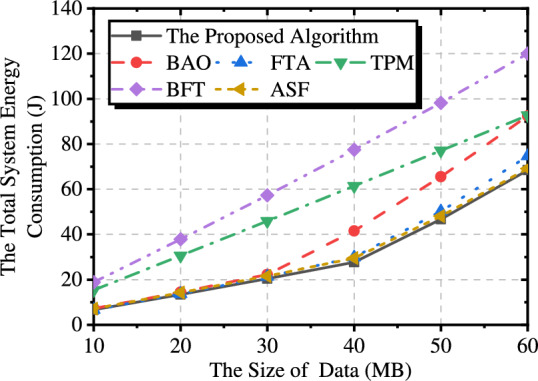
Comparison of methods under different data sizes.

To highlight the performance advantage of our method in reducing system total energy consumption compared with other benchmark methods, we carried out experiments under different data sizes. The experimental results are shown in the Fig. 3. BAO can only offload data to the ship via UAVs, significantly improving performance when the data size is relatively small. This is because the UAVs can leverage sufficient time slots to achieve better transmission channels, thereby reducing energy consumption. However, as the data size increases, the UAVs must stay closer to the buoy to ensure complete data collection. Compared to FTA, which does not optimize the UAVs’ trajectory, our approach demonstrates superior performance, especially under larger data sizes. TPM lacks optimization of the UAVs’ transmission power, resulting in higher energy consumption, particularly during data transfer between UAVs and ships. BFT exhibits the most limited optimization effect, as it only adjusts the offloading amount while keeping other variables at their initial settings. ASF aligns the UAVs’ trajectory with the ship, effectively reducing transmission energy consumption. However, due to the lack of trajectory optimization, its performance still falls short of our approach. In summary, each optimization variable designed in our approach is meaningful, and by optimizing each of them, we are able to progressively approximate the optimal solution.

### Parametric sensitivity analysis

To analyze the sensitivity of the model in this paper to system parameters, we conducted experiments on four key parameters: transmission power of buoy, number of time slots, service coverage of ship, and minimum transmission rate, to comprehensively evaluate the impact of various parameters on the system.

#### Transmission power of Buoy

**Fig. 4:**
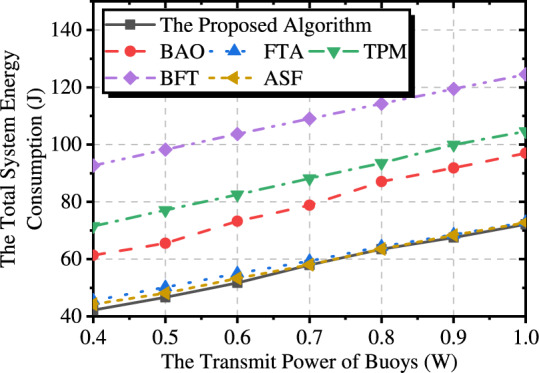
The impact of transmission power of buoy on the system.

Fig. 4 illustrates the total system energy consumption of the different methods under different transmission power of buoys. The results show that the energy consumption of the system increases with transmission power. Moreover, the optimization of the UAVs’ trajectory can only lead to limited performance improvements. This is because the increase in the buoy’s transmission power leads to higher energy consumption for data transfer between the buoy and the UAVs. Even if the UAVs move closer to the buoy to collect data, it is difficult to significantly reduce transmission energy consumption. Although increasing the transmission power of the buoy will increase the transmission energy consumption, an excessively low transmission power would also result in incomplete data collection.

**Fig. 5:**
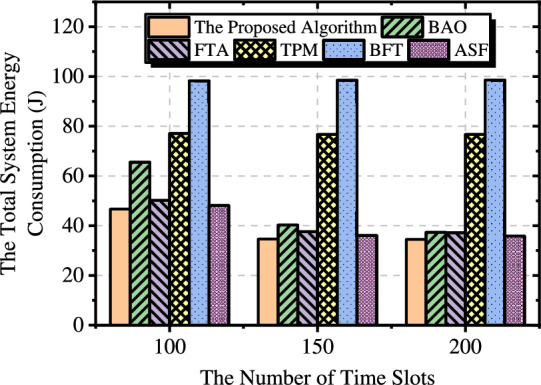
The impact of time slot numbers on the system.

#### Numbers of time slots

Fig. 5 presents a comparison of the total system energy consumption for various methods under different numbers of time slots. The experiment results show that the energy consumption of transmission is negatively correlated with the number of time slots. As the total data size is fixed, an increase in the number of time slots leads to a continuous decrease in energy consumption, eventually reaching a minimum value. BAO can better adjust the trajectory of the UAVs as the number of time slots increases, resulting in the most significant performance improvement. TPM and BFT do not consider optimizing the UAV’s transmission power, so the increase in the number of time slots is balanced by the reduction in transmission delay per time slot, leading to minimal changes in the total system energy consumption.

**Fig. 6:**
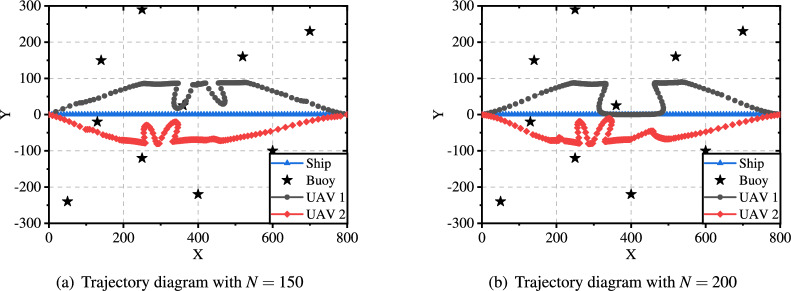
Trajectory diagrams under different numbers of time slots.

Fig. 6 illustrates the flight trajectory of the UAV for 150 and 200 time slots, providing a clear visualization of how the number of time slots affects the trajectory. In comparison to Fig. 11(b), the flight path is longer and more complex. This is attributed to the UAV having more time to complete data collection tasks with increased time slots, enabling it to adopt more intricate flight paths to optimize the data collection process. Furthermore, a comparison between Fig. 6(a) and Fig. 6(b) reveals minimal differences in the trajectories. This suggests that further increases have a negligible impact once the number of time slots exceeds a certain threshold, as the UAV trajectory closely approximates the optimal path under the given conditions.

#### Service coverage of ship

**Fig. 7:**
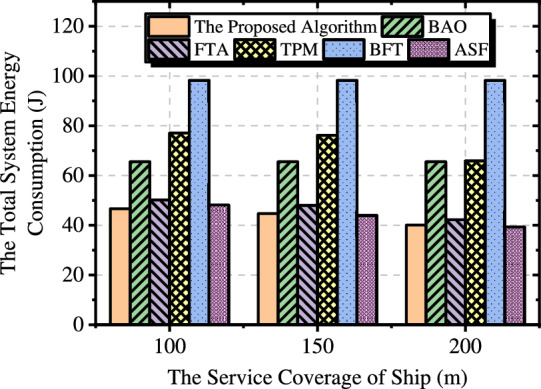
The impact of ship service coverage on the system.

Fig. 7 illustrates the impact of the ship’s service coverage on transmission energy consumption. As the service coverage expands, more buoys can offload data directly to the ship, reducing the additional energy consumption associated with data relays via UAVs. However, the performance of BAO and BFT remains unaffected, as these approaches inherently require data to be offloaded to the ship through UAVs. Additionally, as the service coverage increases, ASF demonstrates a trend of outperforming our approach. This occurs because an expanded service coverage reduces the number of buoys requiring UAV support, enabling the UAV to fly closer to the ship and thereby lowering transmission energy consumption. Nonetheless, our approach continues to achieve superior performance in most scenarios.

**Fig. 8:**
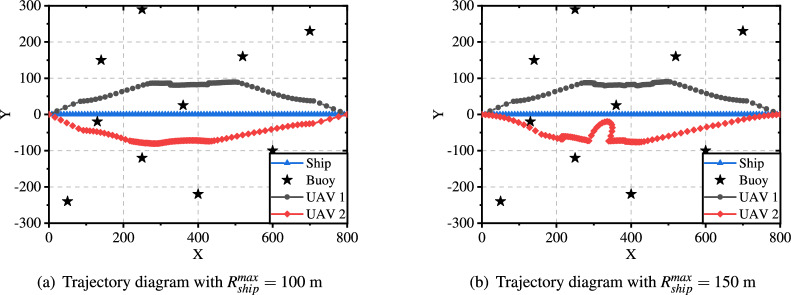
Trajectory diagrams under different service coverage of ship.

**Fig. 9:**
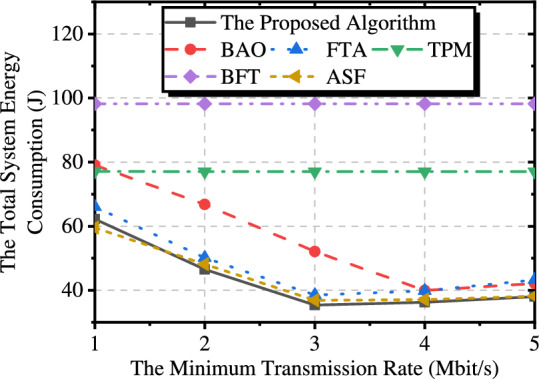
The impact of the minimum transmission rate between UAV and ship on the system

Fig. 8 shows the flight trajectory of the UAV when the service coverage of the ship is set to 100 m and 150 m. Compared with Fig. 11(b) (service coverage was 50 m), the flight trajectory remains basically unchanged when the service coverage is extended to 100 m. This is because the number of buoys in the service coverage of 50 m and 100 m are the same. However, due to the expansion of the service coverage, the buoys within the 100 m range can have more time to offload data, reducing energy consumption (as shown in Fig. 7). When the service coverage is expanded to 150 m, the flight trajectory needs to be adjusted to adapt to the change in the number of buoys for optimal performance.

#### Minimum transmission rate

Fig. 9 shows the effect of the minimum transmission rate between the UAV and the ship on the total system energy consumption. We find that as the transmission rate increases, the total energy consumption tends to decrease initially and then increases. This is because when the transmission rate is low, the UAV may not offload data at certain times, which makes the system fall into a local optimum. However, if the rate is too high, the UAV’s transmission power exceeds the requirements for data offloading, leading to an increase in transmission energy consumption. Therefore, a reasonable minimum transmission rate ensures that the UAV can offload data to the ship evenly throughout the transmission period. TPM and BFT do not optimize the transmit power of the UAV, resulting in the actual transmission rate between the UAV and the ship usually being higher than the set minimum transmission rate. Therefore, they are insensitive to changes in the minimum transmission rate.

In conclusion, the experiments under varying transmission power, number of time slots, and other system settings clearly show that the proposed strategy outperforms other approaches and effectively enhances system performance.

### Convergence analysis

To verify the convergence of our approach, we propose a variety of scenarios with different data collection sizes for the buoys. Specifically, Scenarios 1 to 5 represent data sizes ranging from 10 MB to 50 MB in increments of 10 MB.

**Fig. 10:**
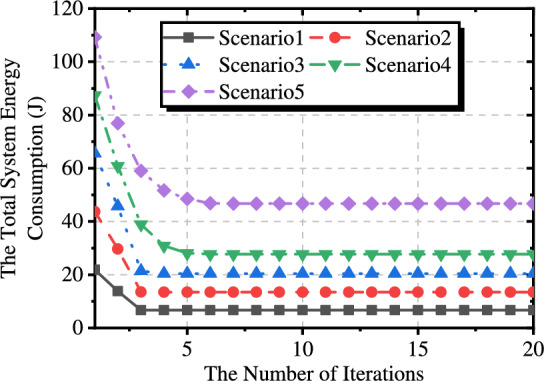
Convergence analysis in different scenarios.

As shown in Fig. 10, our approach can achieve fast convergence in different scenarios, and the number of iterations required increases with the data size increase. Specifically, when the data size is 10 MB, only three iterations are required to meet the accuracy requirements, while when the data size is 50 MB, six iterations are needed.

**Fig. 11:**
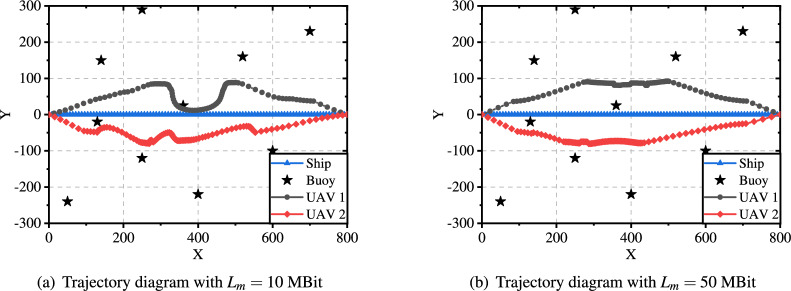
Trajectory diagrams under different data sizes.

Fig. 11 shows the trajectory diagrams under different data sizes (10 MB and 50 MB). When the data size is relatively small, the UAVs fly as close as possible to the ship, and its trajectory fluctuates significantly. This is because the energy caused by data transmission can be effectively reduced when the UAVs are close to the ship. However, as the data size increases to 50 MB, the UAVs need to get closer to the buoy and maintain a stable flight on one side to ensure that all the data can be collected. These experimental results demonstrate that the proposed approach can converge effectively in solving the system energy minimization problem.

## Conclusions

In this paper, we optimized the performance of a dual-UAV-assisted relay marine network, which consists of multiple buoys, two UAVs, and a ship. We aimed to minimize the total system energy consumption by jointly access relationship, UAV trajectory, data offloading, and communication resource allocation. We formulated the problem as a non-convex mixed-integer nonlinear programming problem and proposed a novel approach of alternating optimization. Simulation results demonstrated that the proposed algorithm significantly reduces the total system energy consumption. Future research will explore more complex maritime channel models, broader oceanic regions, and more intricate constraints. Additionally, future work will consider ship motion models, the effects of ocean currents and winds, and the incorporation of low Earth orbit (LEO) satellites.

## Data Availability

The datasets used and/or analysed during the current study available from the corresponding author on reasonable request.
